# Epigenetic rewiring of immunological memory in cancer: heterogeneity, mechanisms, and therapeutic vulnerabilities

**DOI:** 10.3389/fimmu.2026.1890189

**Published:** 2026-10-12

**Authors:** Wenna Liu, Xiaole Pan, Yujia Zhao, Zhimei Li, Dan Wu, Ying Liang

**Affiliations:** 1 Precision Pharmacy & Drug Development Center, Department of Pharmacy, Tangdu Hospital, Fourth Military Medical University, Xi’an, China; 2 Department of Oncology, Tangdu Hospital, Fourth Military Medical University, Xi’an, China; 3 School of Life Science and Technology, Northwestern Polytechnical University, Xi’an, China

**Keywords:** adaptive immunity, cancer, epigenetic editing, immunological memory, tumor immune escape

## Abstract

Immunological memory is a core feature of the immune system that enables rapid and robust responses to re-exposure to the same antigens, underpinning durable antitumor immunity and the efficacy of cancer immunotherapy. However, the epigenetic mechanisms governing the rewiring of immunological memory in the context of cancer—including the heterogeneity of memory immune cells, the regulatory networks of epigenetic modifications, and their therapeutic vulnerabilities—remain incompletely understood. This review focuses on the epigenetic rewiring process of immunological memory (covering both adaptive and innate immune memory) in cancer initiation, progression, and response to therapy, summarizes emerging insights into the heterogeneous characteristics of memory immune cells and their epigenetic regulatory mechanisms, highlights therapeutic opportunities targeting epigenetic rewiring to harness immune memory potential, and discusses the challenges and prospects of translating these findings into clinical practice, aligning with the core objectives of the Research Topic.

## 1 Introduction

Immune memory is the core pillar by which the body resists reinfection by pathogens and eliminates abnormal cells (1–3). In the adaptive immune system, T cells and B cells form long-term memory cells through cloning, expansion, and differentiation (4, 5). In the innate immune system, monocytes, macrophages, and natural killer (NK) cells can also acquire memory-like features known as trained immunity (6, 7). Essentially, the formation of immunological memory relies on durable epigenetic reprogramming (referring to global configurational remodeling across the entire epigenome) (8–10). Epigenetic reprogramming provides a molecular basis for the establishment, maintenance and rapid response of immune memory through stable resetting of DNA methylation profiles, dynamic balance of histone modifications, and reorganization of chromatin three-dimensional conformations (11, 12).

In the tumor microenvironment (TME), infiltrating immune cells deviate from the normal memory differentiation trajectory under the influence of an immunosuppressive milieu and enter a pathologically epigenetic reprogrammed state. T cells are forced to write depletion programs (affecting memory genes such as TCF7, IL7R closed, and inhibitory modified H3K27me3 enrichment), and memory differentiation of B cells is blocked. Innate immune cells tend to develop immune tolerance rather than train immunity (13, 14). The danger of this tumor-induced pathological reprogramming lies in its persistence: Even if the tumor antigen is cleared, the abnormally remodeled epigenome is difficult to recover spontaneously. From a dynamic perspective, physiological immunological memory formation involves epigenetic remodeling (active tuning of chromatin accessibility and 3D conformation) and regulatory network rewiring (reconnection of transcription factor–enhancer–promoter interactions within regulatory circuits) (15). In contrast, these processes are pathologically edited in the TME (here “editing” specifically denotes targeted deposition and erasure of epigenetic marks at defined genomic loci by epigenetic modifiers, which is distinct from DNA sequence-altering genetic editing) to lock cells into exhausted or tolerant phenotypes. Importantly, these pathological modifications leave stable epigenetic scars on the genome—defined as persistent repressive chromatin signatures deposited following chronic aberrant signaling—that constitute a long-standing barrier to the recovery of memory functionality. This is the key reason why, even if the antigen is removed or immune checkpoint inhibitor (ICI) treatment is used, exhausted immune cells still have difficulty fully recovering their memory function (16). Targeted drug intervention can reshape the epigenetic landscape of immune cells, reversing their epigenetic state from exhaustion or tolerance to functional recovery (17, 18) (**Figure 1**).

**Figure 1:**
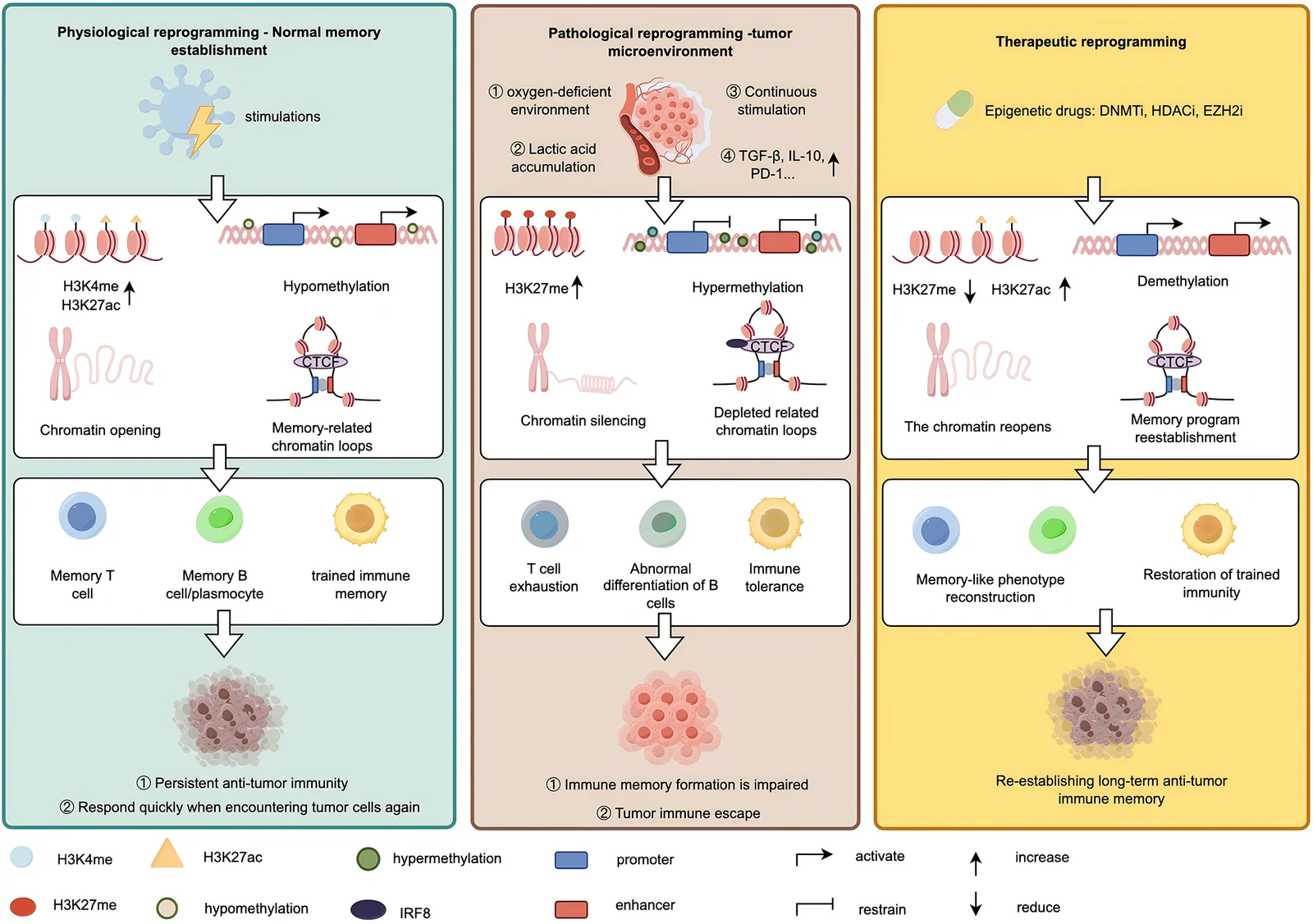
In different states, epigenetic reprogramming confers different epigenetic marks on immune memory, thereby determining the fate of immune cells. Left: Phenotypic reprogramming of physiological state; Middle: Epigenetic reprogramming of the pathological state in the tumor microenvironment; Right: Epigenetic reprogramming after drug intervention.

However, current research is mainly limited to the mechanism of CD8+ T-cell depletion, with limited analysis of B cells and innate immune training memory. Epigenetic drugs carry the risk of off-target effects and inducing autoimmunity, and systematic screening criteria for assessing their therapeutic vulnerability are currently lacking. Clinical translation of combined immunotherapy still lacks reliable biomarkers and administration strategies (19, 20). Of particular note, the efficacy of the aforementioned interventions is constrained by a reversible-to-fixed window, namely the temporal threshold at which aberrant epigenetic modifications transition from a reversible state to a stably fixed state. Interventions administered within this window hold promise for reversing pathological epigenetic programs, whereas epigenetic scars become largely irreversible once this critical window is crossed (21). Accordingly, understanding the heterogeneous map of epigenetic reprogramming of immune memory in cancer, its core molecular mechanisms, and the vulnerable points for intervention provides a theoretical basis for the development of durable, broad-spectrum, and precise antitumor immune strategies.

This review focuses on three core dimensions of cancer immune memory in epigenetic reprogramming: heterogeneity, mechanisms, and treatment of vulnerability. This review systematically combines the current evidence, discusses how to integrate epigenetic interventions into tumor immunotherapy more precisely and safely, and elaborates on the potential value of antitumor immunotherapy based on immune memory as well as the current research challenges and directions.

## 2 Heterogeneity of immunological memory cells in cancer and its epigenetic basis

The heterogeneity of immune memory cells is the structural basis for their ability to cope with the challenges of diverse pathogens. In the context of cancer, this heterogeneity is further shaped by the TME, forming a complex atlas of cell subpopulations. The memory states of T cells and B cells in adaptive immunity and cells with training immune characteristics in innate immunity are defined by a multidimensional epigenetic landscape as a continuous spectrum of differentiation (**Figure 2**; **Table 1**).

**Figure 2:**
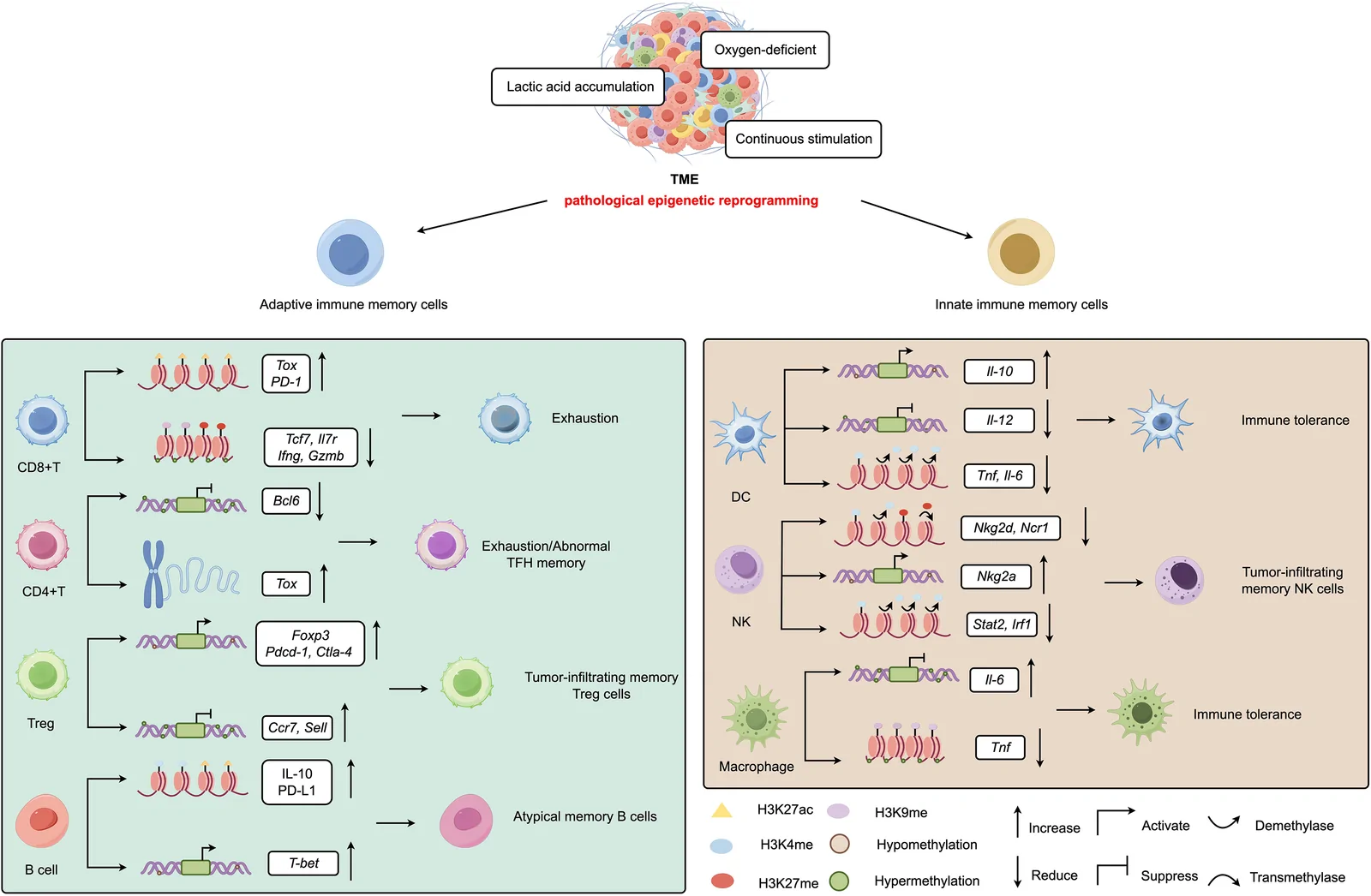
The remodeling of the tumor microenvironment alters the epigenetic tags of various immune memory cell subsets, thereby hindering the formation of immune memory.

**Table 1:** Epigenetic reprogramming of immune cells in tumor microenvironment.

Cell type	Cell state	Key loci	Epigenetic marker type	Epigenetic modification characteristics	References
T cell	Memory T cell	IFNG, GZMB, PRF1, TCF7, CCR7, IL7R, BCL2, EOMES, ZBTB32	DNA methylation, histone modification, chromatin accessibility	DNA demethylation in the promoter regions of IFNG, GZMB, PRF1. Enrichment of H3K4me3/H3K27ac in the enhancers of effector genes. Chromatin opening at IL7R and BCL2 loci. PRC2-mediated H3K27me3 modification of terminal effector genes.	https://pmc.ncbi.nlm.nih.gov/articles/PMC11881328/ https://pmc.ncbi.nlm.nih.gov/articles/PMC8771143/
T cell	Exhausted T cell	PDCD1, LAG3, TIM3, TIGIT, IFNG, TCF1, BCL6, BLIMP1	DNA methylation, histone modification, chromatin accessibility	Sustained increase in chromatin accessibility at PDCD1 locus Decreased chromatin accessibility at IFNG locus. DNA hypermethylation in the promoter regions of TCF1 and BCL6. Enrichment of H3K27me3 in memory-related genes. Demethylation of inhibitory receptor genes.	https://pmc.ncbi.nlm.nih.gov/articles/PMC11881328/, https://pmc.ncbi.nlm.nih.gov/articles/PMC12089470/
B cell	Memory B cell	BLIMP1, BACH2, IRF4, IGHG, IGHA, CD27	DNA methylation, histone modification, epigenetic imprinting	IRF4-mediated epigenetic imprinting of antigen stimulation; Differential histone modification at BLIMP1/BACH2 loci; DNA demethylation at IGHG/IGHA antibody loci; Enrichment of H3K4me3 in re-differentiation-related genes.	https://pmc.ncbi.nlm.nih.gov/articles/PMC8725624/, https://pmc.ncbi.nlm.nih.gov/articles/PMC12289371/
B cell	Immune tolerant B cell	CD22, FCRL4, IL10, TGFB1	DNA methylation, histone modification	DNA hypermethylation in the promoter regions of pro-inflammatory genes. Enrichment of H3K27me3 in activation-related genes. Chromatin closure at IL10 and TGFB1 inhibitory gene loci.	https://pmc.ncbi.nlm.nih.gov/articles/PMC9622816/, https://www.frontiersin.org/journals/immunology/articles/10.3389/fimmu.2025.1575857/full
NK cell	Memory NK cell	PRF1, IFNG, TNF, KIR2DS4, ZEB2, BACH2, TCF7, FCER1G	DNA methylation, histone modification, chromatin accessibility	Enhanced chromatin accessibility at PRF1 locus. DNA demethylation in the promoter regions of IFNG/TNF. Enrichment of H3K4me3/H3K27ac in effector genes. Hypermethylation of FCER1G gene. Demethylation at PRDM1/ZBTB32 loci.	https://pmc.ncbi.nlm.nih.gov/articles/PMC10243875/, https://pmc.ncbi.nlm.nih.gov/articles/PMC12154414/
NK cell	Exhausted NK cell	KIR3DL1, NKG2A, PDCD1, LAG3, IFNG, PRF1	DNA methylation, histone modification, chromatin accessibility	Hypermethylation of IFNG/PRF1 gene loci. Chromatin opening at inhibitory receptor gene loci. Enrichment of H3K9me3 in effector genes. DNA hypermethylation of activation-related transcription factor genes.	https://pmc.ncbi.nlm.nih.gov/articles/PMC11881328/ , https://pmc.ncbi.nlm.nih.gov/articles/PMC8129532/
Dendritic cell (DC)	Activated/Memory DC cell	IRF8, IRF4, PU.1, ID2, BATF3, IL12B, TNF	DNA methylation, histone modification, chromatin accessibility	Chromatin opening at IRF8/IRF4 loci. DNA demethylation in the promoter regions of IL12B and TNF pro-inflammatory genes. Enrichment of H3K4me3/H3K27ac in activation-related genes.	https://pmc.ncbi.nlm.nih.gov/articles/PMC8909611/ , https://www.science.org/doi/abs/10.1126/science.aea1200
Dendritic cell (DC)	Immune tolerant DC cell	IDO1, IL10, TGFB1, PD-L1, IRF8, STAT3	DNA methylation, histone modification, chromatin accessibility	STAT3-mediated DNA hypermethylation + H3K27me3 modification at IRF8 locus. Chromatin opening at IDO1/IL10 loci. Hypermethylation in the promoter regions of pro-inflammatory genes. EZH2-mediated H3K27me3 enrichment.	https://pmc.ncbi.nlm.nih.gov/articles/PMC11075321/ , https://www.preprints.org/manuscript/202604.0868
Macrophage	Trained/Memory macrophage	IL1B, TNF, PTGS2, HIF1A, ATF7, G9a	DNA methylation, histone modification, chromatin accessibility	Enrichment of H3K4me3 in the promoter regions of pro-inflammatory genes. Decreased H3K9me2 modification mediated by ATF7. DNA demethylation at metabolism-related gene loci. Chromatin opening at IL1B and TNF loci.	https://pmc.ncbi.nlm.nih.gov/articles/PMC10193650/ , https://pmc.ncbi.nlm.nih.gov/articles/PMC8615871/
Macrophage	Immune tolerant/Exhausted macrophage	IL10, TGFB1, CD163, MSR1, NFKB1	DNA methylation, histone modification, chromatin accessibility	Enrichment of H3K27me3 in pro-inflammatory gene loci. DNA hypermethylation in the promoter regions of IL1B and TNF. Chromatin opening at IL10/TGFB1 loci. Enrichment of H3K9me3 modification in activation-related genes.	https://pmc.ncbi.nlm.nih.gov/articles/PMC8615871/ , https://pmc.ncbi.nlm.nih.gov/articles/PMC10193650/

### 2.1 Heterogeneity of adaptive immune memory cells and epigenetic rewiring

Adaptive immune memory cells comprise mainly T cells and B cells. T cells have heterogeneous immune memory. Under chronic antigen stimulation, tumor-infiltrating CD8+ T-cells do not simply divide into memory T cells and exhausted T cells (Tex); instead, they present a continuous transitional state from the quiescent-like exhausted precursor cells (Tpex, TCF-1^+^ PD-1^+^ TIM-3^–^) to terminally exhausted cells (Tex_term, TIM-3^+^ TOX^+^) (22–24). Single-cell epigenomics reveals that the chromatin accessibility of Tpex retains some of the characteristics of normal memory T cells: The regulatory elements of memory-related genes such as TCF7 and IL7R remain in an open or semi-open state. Although Tex_term is locked by deeply solidified inhibitory modifications (such as the accumulation of H3K27me3 in the exhaustion-related enhancer regions), its epigenome is closer to terminal differentiation than to quiescent memory (25–27). CD4+ T-cells are also heterogeneous and include tumor-infiltrating Th1 memory, Tfh memory, and regulatory T cells (Tregs), each of which have unique DNA methylation profiles and histone modification patterns. Among them, Tregs are identified by the demethylation of the FoxP3 locus, but their memory phenotype and functional stability are dynamically regulated by signals such as TGF-β in the TME (28).

The heterogeneity of B-cell memory in tumors is complex. Memory B cells, plasmablasts, and long-lived plasma cells have different levels of memory. Tumor-associated B cells (TABs) exhibit a spectrum shift from follicular to regulatory (IL-10^+^ Breg) types, and their epigenetic basis manifests as abnormal methylation of the promoter of antibody class-switching genes (such as AICDA) and chromatin closure at the sites of co-stimulatory molecules (CD40, CD80) (29, 30). The composition of B-cell memory subsets varies according to tumor type. For example, B cells within tertiary lymphoid structures tend to retain a more complete memory epigenetic program, whereas diffuse infiltrating B cells exhibit an inhibitory phenotype (31–33).

In summary, the TME activates epigenetic abnormal reprogramming of adaptive immune cells, leading to the establishment of a complex differentiation map of adaptive immune memory cells. This is key to differences in tumor immune escape.

### 2.2 Heterogeneity of innate trained immunity cells and epigenetic rewiring

The heterogeneity of innate immune memory (trained immunity) is an emerging field (34, 35). Innate immune cells such as monocytes, macrophages, NK cells, and dendritic cells can produce an enhanced response that lasts for several months after being stimulated (36–39). The molecular basis lies in the long-term retention of activating histone modifications (H3K4me3 and H3K27ac) in the enhancer regions of key genes and the epigenetic tags of low DNA methylation (40). In the TME, these cells are reprogrammed to an immune tolerance state, characterized by the deposition of inhibitory histone modifications (H3K27me3) at key gene loci and the disruption of the metabolic-epigenetic feedback loop. For example, in tumor-infiltrating NK cells, activating receptor genes (such as Nkg2d, Ncr1) show enrichment of H3K27me3 and loss of H3K4me3 in the promoter regions. Concurrently, the interferon gamma (IFNG) locus undergoes high DNA methylation, in contrast to the low methylation of normal memory NK cells (41). Different innate immune subsets have different sensitivities to the epigenetic reprogramming induced by tumors: Tissue-resident macrophages are more prone than monocyte-derived macrophages to solidifying the tolerant phenotype, whereas the trained memory of NK cells is more dependent on the synergy of cytokine-induced STAT signaling and epigenetic modifications (42–44).

These epigenetic changes keep memory-like innate immune cells in a state of long-term immune tolerance that is difficult to reverse. This type of pathological trained immunity is an important mechanism for tumor immune escape.

### 2.3 Crosstalk between adaptive and innate memory cells regulated by epigenetic rewiring

The discovery of trained immunity explains the molecular basis of the interaction between innate and adaptive immune memory cells, which is necessary for the overall immune memory system. In the context of tumors, the continuous inhibition of the TME not only prompts pathological reprogramming of adaptive and innate immune memory cells, but also further disrupts the collaborative cooperation of the two major memory systems (45, 46).

Activated T cells can weaken the transcription of hematopoietic prostaglandin D synthase (HPGDS) in tumor-associated macrophages (TAMs) through a TNFα-dependent manner, thereby reversing the tumor-promoting phenotype of TAMs and facilitating the recruitment and activation of CD8+ T-cells. TAMs can inhibit the memory differentiation of T cells through mechanisms such as PD-L1, IL-10, and arginase. The TME determines whether these two cell types cooperate in antitumor or protumor activities by altering the epigenetic landscape of these cells (47, 48).

The two subgroups of memory NK cells that possess adaptability and inherent memory serve as a natural bridge between innate and adaptive immunity. In the ovarian cancer model, NK cells and CD8+ T-cells interact functionally: The absence of either group leads to impairment of the phenotype of the other group. The TME weakens the training memory ability of NK cells and blocks the ability of NK cells to train CD8+ T-cells to differentiate toward a memory direction, causing both defense lines to be paralyzed simultaneously. In addition, immunosuppressive cells in the TME (such as Tregs and myeloid-derived suppressor cells [MDSCs]) constitute the third line of suppression against the crosstalk (43, 49, 50). Epigenetic mechanisms play an important role in regulating the functions of these inhibitory cells.

In summary, during cancer development, the TME drives the epigenetic reprogramming of abnormalities and assists in tumor immune escape. Understanding how the TME reshapes pathological immune memory through epigenetic mechanisms and interferes with crosstalk between immune memory cells is key to identifying therapeutic vulnerabilities and restoring immune memory.

## 3 Core mechanisms of epigenetic rewiring regulating immunological memory in cancer

The core regulatory mechanisms of epigenetic reprogramming include dynamic regulation of DNA methylation, differential changes in histone modifications, regulation of non-coding RNA (ncRNA), and remodeling of chromatin states (30, 51). (**Figure 3**).

**Figure 3:**
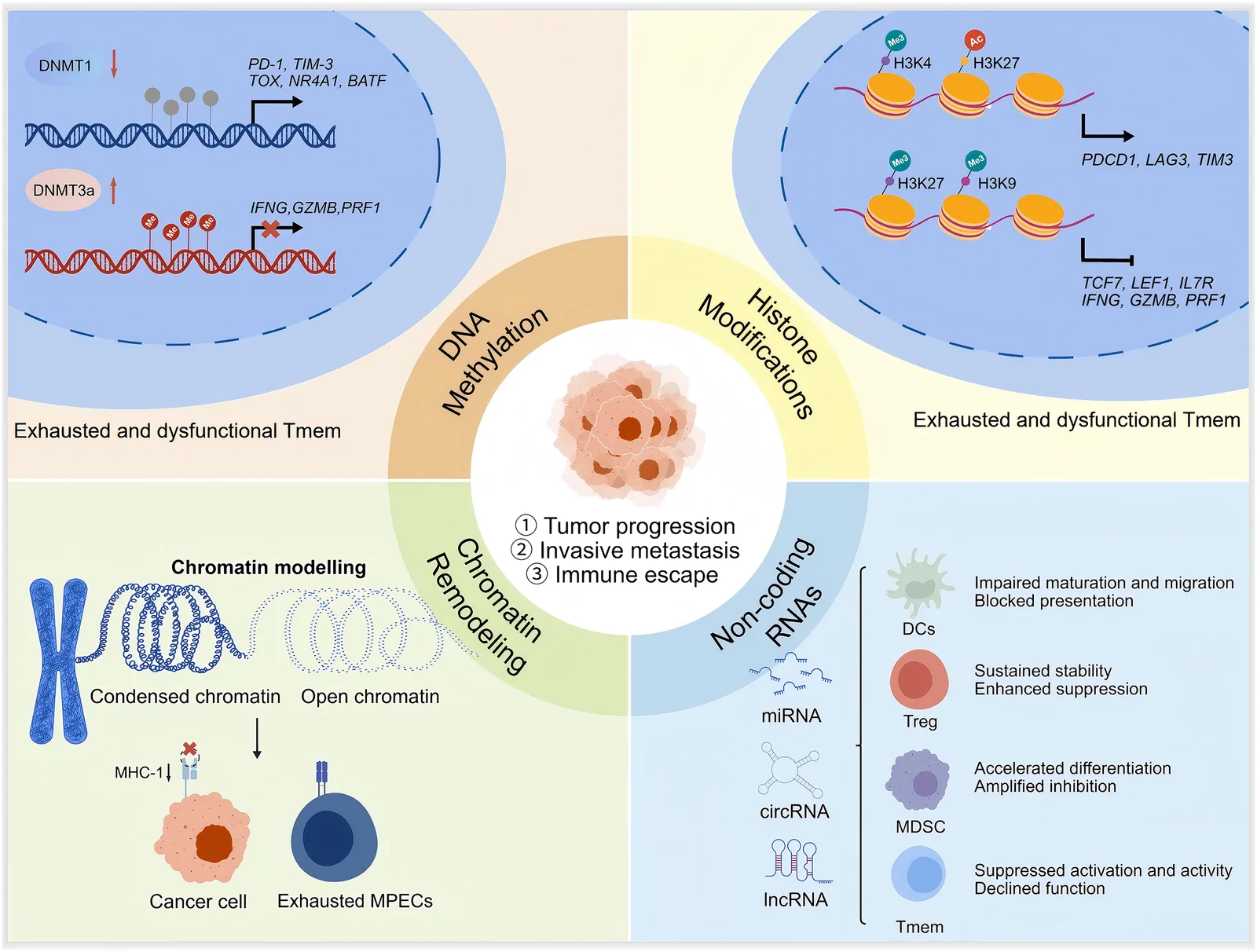
Core mechanisms of epigenetic rewiring of immune memory in cancer. This figure summarizes the major epigenetic remodeling mechanisms involved in the regulation of immune memory. These mechanisms are presented sequentially, including DNA methylation/demethylation, histone modifications, non-coding RNA-mediated regulation, and the remodeling of chromatin states, with a brief overview of their functional and regulatory roles. DNMT1 DNA methyltransferase 1, DNMT3a DNA methyltransferase 3A, Tmem memory T cells, MPECs memory precursor effector cells.

### 3.1 DNA methylation/demethylation rewiring: silencing and activation of memory-related genes

The formation and maintenance of immune memory are closely related to the dynamic changes of DNA methylation/demethylation. Under physiological conditions, this equilibrium is coordinately maintained by DNMT1 (responsible for preserving post-replicative methylation patterns), DNMT3A (mediating *de novo* methylation), and the TET enzyme family (catalyzing active demethylation) (52–54).

In adaptive immune cells, the promoters of effector genes (Ifng, Gzmb, Prf1) remain hypomethylated, while their enhancers are predominantly marked by 5hmC modifications. The promoters of inhibitory receptor genes (Pdcd1, Ctla4) are kept hypermethylated. Promoters and enhancers of memory stemness genes (Tcf7, Bcl6, Sell) exhibit hypomethylation, accompanied by hypermethylation within gene bodies, which guarantees the proper differentiation and long-term survival of memory cells (40, 54, 55). In innate immune cells, TET-dependent demethylation (conversion of 5mC to 5hmC) occurs at the enhancers of pro-inflammatory genes (Tnf, Il6, Il1b), establishing long-term functional potentiation effects of trained immunity (53).

Within the tumor microenvironment (TME), persistent antigen stimulation disrupts the DNMT-TET balance. Upregulated DNMT3A triggers aberrant *de novo* methylation at the promoters of effector genes. Meanwhile, reactive oxygen species (ROS) accumulation and α-ketoglutarate (α-KG) depletion suppress TET activity, impairing its capacity to eliminate checkpoint gene promoters (Pdcd1, Lag3, TIGIT) via active demethylation. This leads to aberrant hypomethylation and transcriptional activation of these checkpoint genes. Additionally, rapid T cell proliferation upon chronic stimulation results in insufficient maintenance methylation by DNMT1, causing replication-coupled passive demethylation and further aggravating methylation loss at checkpoint loci. Conversely, insufficient TET activity leads to sustained hypomethylation at the promoters of master exhaustion transcription factors (Tox, Nr4a1, Batf), alongside abnormally reduced 5mC levels in their gene bodies (a pattern opposite to normal memory cells). This drives their constitutive overexpression. Ultimately, multi-layered epigenetic locking—coupled with the enrichment of H3K27me3 at the promoters of memory genes and other events—establishes irreversible epigenetic scars characteristic of T cell exhaustion (56–58).

In summary, aberrant DNA methylation reprogramming is key to the exhaustion and dysfunction of immune memory during the malignant progression of cancer.

### 3.2 Histone modification rewiring: remodeling chromatin state of memory immune cells

Another important epigenetic mechanism related to immune memory is histone modification. In the process of immune memory establishment, the promoter and enhancer regions of memory stem genes (TCF7, LEF1, and IL7R) and effector genes (IFNG, GZMB, and PRF1) are mainly enriched in activating modifications such as H3K4me3 and H3K27ac, which lead to chromatin opening and promote gene transcription. However, typical repressive modifications such as H3K27me3 and H3K9me3 are enriched in the promoter regions of inhibitory receptor genes (PDCD1, LAG3, and TIM3) and terminal exhaustion-related genes, mediating chromatin condensation and silencing gene transcription. In addition, histone phosphorylation (such as H3S10ph, H3S28ph), ubiquitination (such as H2BK120ub) and other modifications are also involved in the regulation of the chromatin state of memory immune cells, forming a cooperative regulatory network (14, 40, 59–62). Nevertheless, the regulatory effects of the aforementioned modifications are not absolute. The same epigenetic mark can exert opposing regulatory roles depending on genomic loci, immune cell subsets, or cellular differentiation status. For instance, H3K27me3 is also involved in the activation of tissue-specific genes at certain enhancer regions. In addition, histone phosphorylation, ubiquitination, and multiple newly identified acylation modifications including crotonylation, β-hydroxybutyrylation and lactylation collectively modulate chromatin states in memory immune cells, jointly assembling an intricate synergistic regulatory network (30, 63).

In tumors, the abnormal signal of the TME can break the dynamic balance of normal histone modification, leading to histone modification programming, restoration of the immune cell chromatin state, or drives their differentiation toward exhausted or tolerant phenotypes. Abnormal histone reprogramming involves changes in modifications such as methylation, acetylation, phosphorylation, and ubiquitination. Their functional outcomes vary depending on modification sites, cell subsets and tumor types, predominantly manifesting as transcriptional silencing of effector and stemness genes alongside aberrant upregulation of inhibitory genes, ultimately locking immune cells into a functionally impaired state (51, 64).

### 3.3 Non-coding RNA-mediated epigenetic rewiring: post-transcriptional regulation of immunological memory

In addition to the epigenetic regulation of DNA methylation and histone modification, ncRNA regulation collaboratively participates in the formation and long-term maintenance of immune memory (9, 65). For example, miR-379-5p inhibits immune checkpoints (TIM-3 and TIGIT) to promote the differentiation of CD8+ T-cells into memory-like cells and restore their killing function, whereas the inhibitor of miR-1290 can activate the c-Jun N-terminal kinase (JNK) signaling pathway and enhance the immune response of tissue-resident memory T cells (TRMs) (66). In addition, long non-coding RNA (lncRNA) Malat1 binds to the miR-15/16 family, indirectly promoting the expression of genes such as CD28, which is crucial for the activation and formation of memory in T cells (67).

Furthermore, certain circular RNAs can activate innate immune pathways such as RIG-I, and reverse immune suppression (68). Multiple types of RNA modifications such as m6A and m5C are involved in regulating the differentiation and function of immune cells (69, 70). These ncRNAs are interwoven with RNA modifications, jointly forming a comprehensive regulatory network covering transcription, post-transcription, translation, and post-translational modifications (68, 71).

Influenced by the TME, the abnormal regulation of epigenetic reprogramming mediated by ncRNAs can promote tumor immune escape through various means, such as inhibition of antigen presentation, formation of immunosuppressive microenvironments, and dysfunction of immune cells. For example, miR-155 can inhibit the expression of CCR7, damaging the maturation and migration of dendritic cells, resulting in them being unable to effectively present tumor antigens, and inhibiting the activation of memory T cells (72). Exosomal miR-27a-3p can be secreted by tumor cells into the TME, upregulating the expression of PD-L1 on the surface of immune cells, inhibiting the activity of memory T cells (73). Tregs through ncRNA-mediated demethylation of FOXP3 maintain their stability, promoting immune suppression (74). lncRNA nicotinamide N-methyltransferase (NNMT) can reduce H3K27me3 modification, promoting the expression of granulocyte-macrophage colony-stimulating factor, differentiation of MDSCs, and inhibiting antitumor immunity (75).

In summary, the regulation of ncRNA and RNA modification enables the control of immune memory over tumor immune responses to be more precise.

### 3.4 Chromatin remodeling and 3D genome architecture rewiring

The regulation of the functional fate of immune cells in tumors is not only achieved through local epigenetic modifications but also involves changes in the chromatin landscape. Epigenetic reprogramming-mediated chromatin landscape remodeling is mainly regulated by chromatin remodeling complexes and changes in the three-dimensional genome structure (76–78). For example, in the differentiation of CD8^+^ T-cells, the BAF complex promotes the formation of the epigenetic landscape associated with effector and memory of CD8+ T-cells. In memory T-cells, transcription factors such as SOX4 and NRF2 maintain the expression of their target genes, in conjunction with the action of chromatin remodeling complexes, thereby supporting the epigenetic reprogramming of memory cells. Tcf1 regulates the three-dimensional genome to preprogram the transcriptional enhancers of functional genes, mobilizing the ability to activate gene expression after the secondary response (79–82).

In a pathological environment, the TME drives immune escape at the spatial conformational level by reshaping the chromatin landscape and the three-dimensional genome structure. Tumor cells use mechanisms such as super enhancers, and additional chromosomal DNA to disrupt the chromatin loops of major histocompatibility complex (MHC) genes or reconfigure the enhancer network, thereby silencing antigen presentation (83, 84). Concurrently, continuous antigen signals in the TME induce the formation of IRF8-dependent CTCF chromatin loops in CD8+ T-cells, locking memory precursor cells into an exhausted phenotype (82, 85, 86).

In summary, the continuous antigen exposure and inhibitory signals in TME force the chromatin spatial conformation to shift from the memory label to the exhaustion label. This three-dimensional genomic-level epigenetic memory is more persistent and harder to reverse than one-dimensional epigenetic modifications.

## 4 Epigenetic reprogramming of immune memory in cancer progression and immunotherapy response

During tumorigenesis, epigenetic reprogramming of immune memory unfolds across both temporal and spatial dimensions. In early lesions chromatin plasticity gradually consolidates into fixed epigenetic configurations in advanced disease. Spatially, the TME establishes discrete immune niches that reinforce T-cell exhaustion through intercellular crosstalk and metabolic competition. These coordinated changes impair antitumor immunity, restrict the effectiveness of immunotherapy, and enable chromatin-based patient stratification. Epigenetic reprogramming during tumor initiation and progression undermines immune memory, fixed chromatin states drive immunotherapy resistance, and epigenetic signatures serve as predictive biomarkers. Collectively, these mechanisms establish epigenetic regulation of immune memory as a core framework for understanding tumor-immune co-evolution (**Figure 4**).

**Figure 4:**
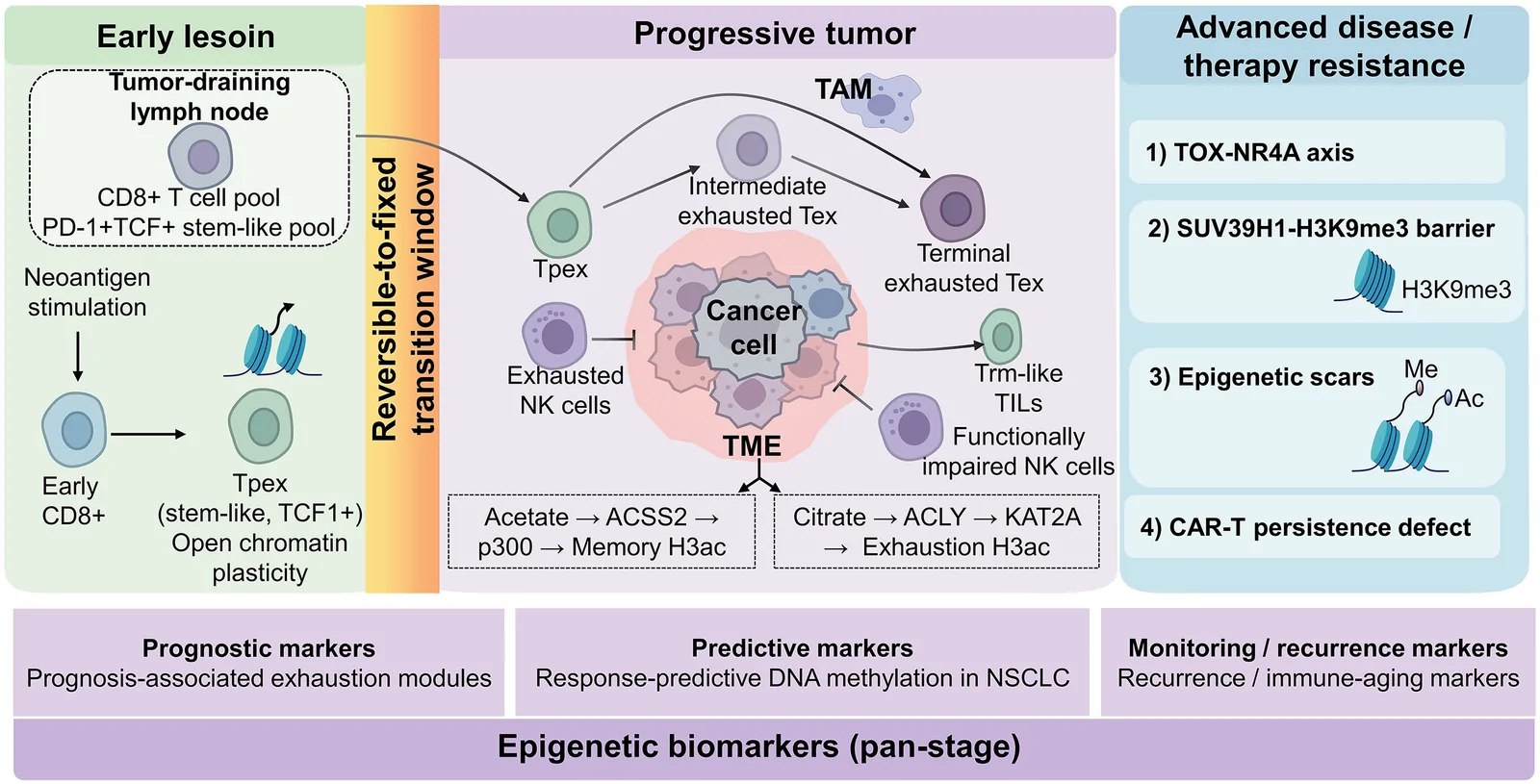
Epigenetic reprogramming of immune memory in cancer progression. (Left) In early lesions, neoantigen stimulation drives clonal expansion of CD8^+^ T cells, which acquire a stem-like exhausted phenotype (Tpex, TCF1^+^PD-1^+^) with open chromatin plasticity. The tumor-draining lymph node harbors a self-renewing Tpex reservoir that undergoes directional recruitment to the tumor. (Center) Progressive tumors undergo reversible-to-fixed transition via metabolic competition (acetate/ACSS2/p300 versus citrate/ACLY/KAT2A) and TAM (tumor-associated macrophage) antigen presentation, generating Tex_term (terminally exhausted T cells) impaired TRM (tissue-resident memory)-like TILs (tumor-infiltrating lymphocytes) and impaired NK cells. (Right) In advanced disease and immunotherapy resistance, four convergent epigenetic mechanisms consolidate fixed exhausted states: (1) the TOX–NR4A transcriptional axis reinforces exhaustion; (2) SUV39H1-mediated H3K9me3 establishes a repressive chromatin barrier; (3) epigenetic scars, including DNA methylation, persist after antigen withdrawal; and (4) aberrant gene methylation impairs CAR-T cell persistence. (Bottom) Epigenetic biomarkers span all disease stages: prognosis-associated exhaustion modules, response-predictive DNA methylation signatures (such as in non-small cell lung cancer (NSCLC), and monitoring markers for recurrence and immune aging. TME, tumor microenvironment; solid arrows, direct transitions; T-bars, inhibition.

### 4.1 Epigenetic reprogramming during tumor initiation and progression

Epigenetic reprogramming of immune memory is a central mechanism of tumor immune evasion. During early tumorigenesis, malignant transformation reshapes the TME. This remodeling alters chromatin landscapes in both adaptive and innate memory immune cells, directly impairing their antitumor functions.

After tumor onset, neoantigens trigger primary adaptive immune responses and drive clonal expansion of antigen-specific CD8+ T-cells. However, persistent antigen stimulation induces progressive chromatin remodeling that redirects effector T cells toward exhaustion. Tex acquire stable epigenetic scars that persist even after antigen clearance, blocking their differentiation into functional memory T cells (25). Such scars include sustained DNA hypermethylation at the Tcf7 promoter and repressive chromatin states at memory-associated loci. Even after withdrawal of chronic stimulation, recovered Exhausted T cells only regain partial memory-like phenotypes, while their epigenomic profiles remain dominated by exhaustion signatures (87, 88). Four distinct subsets of exhausted CD8^+^ T cells have been characterized to date: progenitor exhausted T cells (Tpex), intermediate exhausted Tex, terminally exhausted Tex (Tex_term), and memory-like Tex, each endowed with unique transcriptional and epigenetic features (89). Tpex cells, marked by TCF1 expression and stemness, act as a proliferative reservoir. Chronic antigen exposure epigenetically silences stemness genes, driving terminal differentiation into dysfunctional effectors (89). Epigenetic regulators governing clonal hematopoiesis (DNMT3A, TET2, ASXL1) control the stemness of CD8^+^ T cells during immunotherapy, and their knockout enables the preservation of the Tpex pool (62). This reflects the role of these factors in T-cell epigenetic regulation and does not imply that their knockout in T cells eliminates malignant transformation in hematopoietic stem cells. Recent viral infection models demonstrate that precursors of Tpex are pre-emptively formed during the early phase of acute infection, challenging the conventional view that these precursors are generated exclusively in chronic infections or tumours (90, 91). However, the above observations are primarily obtained from LCMV models. Their generalizability in tumor settings remains to be validated, and it is unclear whether their epigenetic status differs from that of their chronic-phase counterparts.

Memory differentiation of B cells is also finely orchestrated by epigenetic programs. The establishment of memory B cells (MBCs) involves epigenetic remodeling at the loci encoding master transcription factors such as BCL6 and PRDM1, including enrichment of H3K4me2/H3K27Ac and reconfiguration of methylation patterns at the BCL6 promoter (92). Moreover, the expression and targeting specificity of AID during antibody class switching and affinity maturation are stringently constrained by DNA methylation and histone modifications. Within the TME, tumor-infiltrating B cells frequently exhibit dysfunctional phenotypes, manifested as either sustained or aberrant silencing of BCL6, downregulated CD27, and polarization toward IL-10^+^ regulatory B cells (Bregs). These alterations are accompanied by an imbalanced ratio of H3K27me3 to H3K4me3 at the BCL6 locus and abnormal accumulation of H3K4me3 at the IL10 promoter. In addition, off-target AID activity correlates with promoter hypermethylation of DNA repair genes, which may exacerbate genomic instability in B cell populations (93, 94). For innate immunity, trained immunity in NK cells is established via pre-stimulation with IL-12/IL-18, characterized by H3K4me3 enrichment at the promoters of Ifng and Tnf (95). By contrast, tumor-associated NK cell dysfunction is coupled with hypermethylation of the NKG2D promoter and reduced chromatin accessibility at the granzyme B locus (96). Trained immunity in monocytes/macrophages can be induced by β-glucan or BCG, which establishes persistent open chromatin at Tnf, Il6 and other proinflammatory loci. Tumor-associated macrophages (TAMs) undergo M2-like polarization, accompanied by promoter hypermethylation of Tnf/Il12 and H3K27Ac enrichment at the Arg1 and Il10 promoters (6, 97). Myeloid-derived suppressor cells (MDSCs) harbor a pathological epigenetic landscape in the TME, featuring robust H3K27Ac enrichment and DNA hypomethylation at *Arg1* and *Nos2*, as well as aberrant demethylation of CpG islands within the *S100a8/a9* promoter region (98).

Tissue-resident memory T cells (TRMs) play a vital role in tumor surveillance and display tissue-specific transcriptional, epigenetic and functional adaptations. Their metabolic programs (fatty acid oxidation, oxidative phosphorylation) are tightly coupled with epigenetic status to sustain tissue residency and effector capacity (99, 100). In the TME, TRM-like tumor-infiltrating lymphocytes (TILs) are associated with favorable prognosis, whereas tumor-derived signals can remodel their epigenetic programs and cause functional impairment (101).

Extrinsic TME signals also actively redirect epigenetic trajectories toward exhaustion. Innate immune cells with memory-like properties, such as macrophages and NK cells, undergo parallel epigenetic reprogramming, losing surveillance functions via chromatin-mediated silencing of effector genes (36, 43, 102). This convergent dysregulation of adaptive and innate immunity establishes an immunosuppressive niche that accelerates immune evasion.

Convergent epigenetic dysregulation across adaptive and innate immune compartments collectively constructs an immunosuppressive niche that accelerates tumor immune evasion. The above conclusions are largely based on correlative analyses from mouse tumor models and human tumor specimens, necessitating further lineage tracing and functional validation experiments for definitive confirmation.

Beyond immune cells, intrinsic epigenetic alterations in tumor cells themselves make substantial contributions to immune escape. Tumor cells can silence tumor antigens or major histocompatibility complex class I (MHC-I) antigen processing and presentation genes (B2M, TAP1) via aberrant promoter CpG island hypermethylation, thereby evading immune recognition (103). Meanwhile, epigenetic regulation driving PD-L1 upregulation in tumor cells, exemplified by super-enhancer activation and demethylation at the PD-L1 promoter, strengthens the transmission of immunosuppressive signals (104). These cell-intrinsic epigenetic aberrations in malignant cells synergize with pathological epigenetic reprogramming in immune cells to jointly drive the immunosuppressive polarization of the TME.

In addition to cell-intrinsic residency programs, microenvironmental metabolic factors critically shape the epigenetic states of TILs. Nutrient availability directly influences histone modifications governing the fate of T cells. Ma et al. demonstrated that exhausted CD8+ T-cells undergo a metabolic switch from acetate to citrate metabolism, mediated by differential regulation of ACSS2 and ACLY. This nutrient-driven histone modification, involving KAT2A-ACLY and p300-ACSS2 complexes, determines whether T cells maintain effector/memory potential or commit to exhaustion programs. Specifically, acetate-derived acetyl-CoA promotes memory-associated histone acetylation via p300-ACSS2, whereas citrate metabolism through KAT2A-ACLY promotes exhaustion-specific chromatin modifications (105). This provides a direct mechanistic link between TME metabolic competition and chromatin remodeling, providing a rationale for metabolic-epigenetic combination therapies.

Furthermore, TAMs promote the transition of T cells from Tpex to Tex_term via antigen presentation, forming a spatiotemporal regulatory crosstalk between myeloid and lymphoid compartments (102, 106). Lactate-mediated histone lactylation reprograms the immunophenotype of tumor cells and, through epigenetic remodeling, promotes microglia/macrophage polarization toward an M2-like immunosuppressive phenotype, thereby forming a metabolic–epigenetic–immunosuppressive loop. Obesity induces PD-1 expression on macrophages through a metabolic-epigenetic axis (107).

In summary, epigenetic reprogramming imposes stable scars on Tex cells, depletes stem-like precursors, and remodels both adaptive and innate immunity. Although early-stage tumors retain chromatin plasticity and are amenable to metabolic or immunologic intervention, advanced tumors progressively consolidate these epigenetic scars into fixed configurations. This transition from reversible to fixed chromatin states has profound implications for the effectiveness of immunotherapy, as discussed below.

### 4.2 Epigenetic reprogramming and immunotherapy resistance

The chromatin states of exhausted immune cells constitute a major barrier to effective immunotherapy. Consistent with the transition from reversible plasticity to fixed configurations, advanced tumors exhibit exhaustion programs that resist pharmacologic reversal. Although epigenetic alterations progress in a stage-dependent manner, resistance often emerges after an initial response via selection of cells with pre-established, fixed exhaustion programs that resist pharmacologic reversal.

Aberrant epigenetic remodeling of immune memory is a core mechanism of resistance to ICIs, chimeric antigen receptor (CAR) T-cell therapy, and cancer vaccines. The chromatin stability of Tex directly limits the durability of reinvigoration. Repressive chromatin states at memory and effector loci remain largely unchanged after PD-1 blockade (108), and Tex exhibit unique chromatin accessibility profiles distinct from those of naive, effector, and memory T cells (109). Checkpoint blockade alone cannot reverse deeply fixed exhaustion programs, explaining the frequent relapse after an initial response. This stability is actively maintained by chromatin-modifying complexes, even in the absence of continuous antigen signaling.

The transcription factor, TOX, acts as a master epigenetic regulator that enforces T-cell exhaustion and mediates resistance to checkpoint reinvigoration. TOX recruits chromatin modifiers to establish repressive states at memory loci (22) and stabilizes the Tex phenotype (110). Sustained TOX expression is essential for maintaining the epigenetic fate of exhausted CD8+ T-cells. TOX ablation drives chromatin remodeling toward an effector state. The TOX-centered epigenetic axis fundamentally limits the effectiveness of immunotherapy, and the chromatin environment shaped by TOX is resistant to PD-1/PD-L1 blockade-mediated reprogramming (27). TOX and the NR4A family are convergent nodes in the transcriptional-epigenetic control of exhaustion, with NR4A factors reinforcing the exhausted state by cooperating with TOX to suppress effector gene accessibility (22, 111).

SUV39H1-mediated H3K9 methylation is an additional epigenetic barrier to immunotherapy. In colon cancer, SUV39H1 represses cytotoxic T-lymphocyte effector genes, promoting immune evasion (112). In melanoma, SUV39H1 epigenetically regulates CD8+ T-cell responsiveness to anti-PD-1 therapy (113). In adoptive cell therapy, disruption of SUV39H1-mediated H3K9me3 sustains CAR T-cell function in hematologic malignancies (114) and enhances long-term CAR T function in solid tumor models (115). These convergent findings establish SUV39H1 as a potential drug target in multiple immunotherapy modalities.

Epigenetic resistance mechanisms operate distinctly from classical resistance pathways such as low tumor mutational burden (TMB), antigen loss, or defective antigen presentation. Although classical resistance involves genetic alterations or antigenic escape, epigenetic resistance reflects stable chromatin configurations that persist even when antigen recognition remains intact. These pathways may coexist: Tumors with high TMB can still exhibit epigenetic exhaustion, and epigenetic priming may accelerate antigen-loss variants.

Similar epigenetic barriers operate in adoptive cell therapies. CAR T-cell expansion and persistence depend on their epigenetic state at infusion. Chronic antigen stimulation drives DNMT3A-mediated *de novo* DNA methylation at the Tcf7, Eomes, and Tbx21 loci during T cell exhaustion, establishing an irreversible epigenetic scar that is refractory to anti-PD-L1 monotherapy and constrains the efficacy of both checkpoint blockade and CAR-T cell therapy. Targeted inhibition or genetic ablation of DNMT3A synergistically enhances T cell function, identifying it as a critical target for engineered T cell therapies. CAR T-cells often adopt exhaustion programs similar to endogenous TILs (116). PD-1+TCF1+ CD8+ T-cells represent a reservoir population with a distinct epigenetic signature, and this subset constitutes the exclusive source of the proliferative burst following PD-1 blockade during chronic infection (117). STAT5 antagonizes TOX and rewires Tex toward durable effector-like states, whereas BATF and IRF4 cooperate to counteract CAR T-cell exhaustion (118, 119). These findings establish the epigenetic state of infused T cells as a critical determinant of CAR T-cell therapy outcomes.

Recognition of these barriers has spurred research into epigenetic-targeted drugs. DNMT inhibitors reverse T-cell exhaustion through demethylation of memory gene promoters, and histone deacetylase (HDAC) inhibitors modulate chromatin accessibility to favor effector states (120). However, first-generation epigenetic agents face substantial translational obstacles. In hematologic malignancies, azacitidine monotherapy in older patients with acute myeloid leukemia (AML) failed to demonstrate superior event-free survival compared with conventional care regimens (121). In solid tumors, dose-limiting toxicities including myelosuppression, fatigue, and gastrointestinal adverse events, restrict the maximum tolerated dose of broad-spectrum DNMT and HDAC inhibitors, narrowing the therapeutic window (122). These limitations explain why epigenetic monotherapy has not achieved broad regulatory approval for use in combination with immunotherapy and highlight the need for biomarker-guided patient stratification to identify potential responders.

Collectively, fixed epigenetic states of Tex constitute a primary resistance mechanism across multiple immunotherapy modalities, including ICIs, CAR T-cell therapy, and cancer vaccines. The TOX-NR4A axis and SUV39H1-mediated H3K9 methylation actively maintain these states, rendering them refractory to checkpoint blockade alone and necessitating epigenetic-targeted combination strategies.

### 4.3 Epigenetic signatures as predictors of the effectiveness of immunotherapy

Having established that fixed epigenetic states constitute a primary resistance mechanism to multiple immunotherapy modalities, the ability to identify these states prior to therapy, and to monitor their dynamics during therapy, is critical for patient stratification and therapeutic optimization. Translating these mechanistic insights into clinical practice requires robust predictive biomarkers. Recent advances in epigenetic profiling have identified chromatin signatures associated with therapeutic outcomes, offering new opportunities for patient stratification. These biomarkers can be broadly classified into three categories: (1) prognosis-associated signatures (such as exhaustion modules in estrogen receptor [ER]-positive breast cancer), (2) response-predictive chromatin states (such as DNA methylation patterns in non-small cell lung cancer [NSCLC]), and (3) monitoring markers for immune aging or recurrence.

Pan-cancer multi-omics studies have uncovered epigenetic immune memory biomarkers linked to treatment response. Human T-cell differentiation atlases have defined functional enhancers that distinguish responsive from non-responsive states (123). Combined LAG-3 and PD-1 blockade induces co-expression of cytotoxic and exhaustion gene modules whose balance predicts antitumor immunity (124). Exhaustion signatures predict response in ER-positive breast cancer and thyroid cancer (125, 126).

The chromatin signatures of TILs have the potential to predict effectiveness at single-cell resolution. DNA methylation-based profiling predicts anti-PD-1 response in NSCLC, outperforming traditional biomarkers (127). Single-cell landscapes have revealed conserved and cancer-type-specific epigenetic programs (128). Neoantigen-specific TIL transcriptional programs can distinguish responders from non-responders (129).

Spatial epigenomic analysis holds great promise as a robust tool to dissect the tumor-immune interface. Zhu et al. used spatial and multi-omics analysis of lung adenocarcinoma precursors to reveal TIM-3-high features enriched in precancerous lesions, identifying TIM-3 as a putative target for precancer interception (130). This stage-dependent spatial architecture highlights the importance of integrating epigenetic signatures with spatial context for precise patient stratification.

Epigenetic signatures also forecast recurrence, prognosis, and immune aging. Spatial HLA signatures predict response in renal cell carcinoma (131). Stem-like CD8+ T-cell pools in tumor-draining lymph nodes sustain antitumor immunity, and their chromatin status predicts long-term control (132). Epigenomic-guided mass cytometry profiling identifies exhaustion subtypes with distinct prognostic implications (133).

Clinical implementation faces several challenges: interlaboratory batch effects, need for standardized workflows, and computational complexity. Emerging solutions include reference epigenomes for batch correction, machine learning frameworks for integrative analysis, and prospective validation cohorts. Integrating epigenetic signatures with PD-L1 expression, TMB, and microsatellite instability (MSI) improves predictive accuracy. Epigenetic biomarkers offer unique advantages, such as dynamic responsiveness to TME changes, that complement rather than replace existing markers. The ultimate goal is to intervene during the reversible-to-fixed transition window to reverse pathological chromatin states.

In summary, multi-omics and single-cell epigenetic profiling analyses provide a vital strategy for screening robust biomarkers predictive of immunotherapy response. From a translational perspective, a noteworthy research direction lies in defining the time window during which epigenetic states transit from reversible to permanently fixed configurations, so as to lay a theoretical basis for timely therapeutic intervention. Clinical implementation hinges on the establishment of standardized experimental protocols and their integrated validation alongside existing biomarkers to enable precise patient stratification. The above mechanistic investigations reveal the dual nature of epigenetic reprogramming in tumor regulation: it drives tumor immune evasion and therapeutic resistance on one hand, yet its stage-dependent characteristics may offer actionable windows for targeted intervention on the other. In our opinion, translating these insights into clinical practice requires strategies that (1) reverse fixed exhaustion programs, (2) prime the immune system for enhanced effectiveness of checkpoint blockade, and (3) engineer immune cells with durable memory potential. The validity of this postulation remains to be verified in clinical cohorts.

## 5 Therapeutic vulnerabilities: targeting epigenetic reprogramming of immune memory

This section discusses three complementary therapeutic strategies: (1) pharmacologic inhibition of chromatin-modifying enzymes, (2) rational combination with immunotherapy, and (3) precision engineering of immune cells. A central translational challenge is to target the reversible-to-fixed transition window before chromatin states become irreversibly fixed (**Figure 5**; **Table 2**).

**Figure 5:**
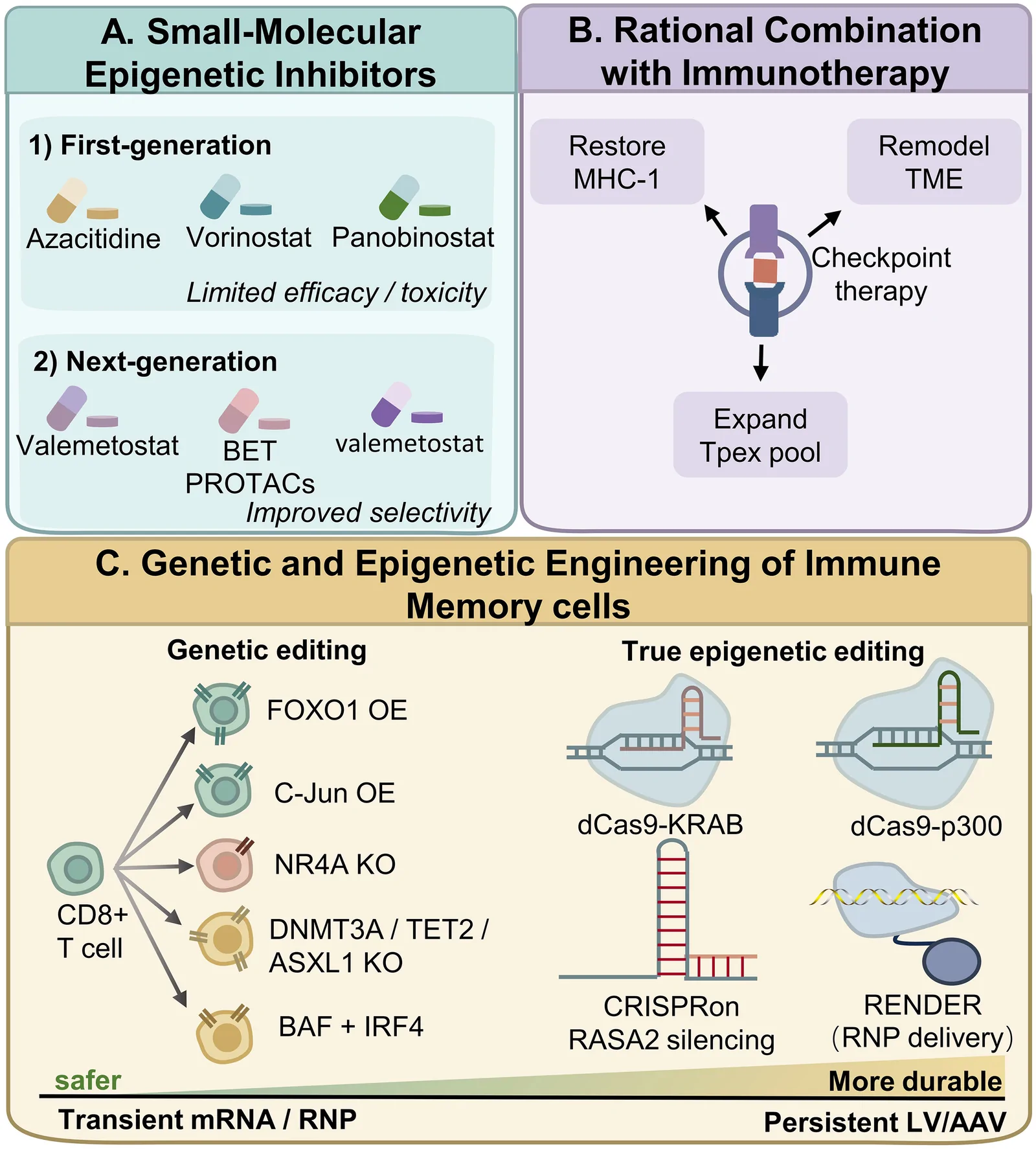
Therapeutic strategies targeting epigenetic reprogramming of immune memory. Three complementary strategies exploit the reversible-to-fixed transition window. **(A)** Small-molecule epigenetic inhibitors include first-generation broad-spectrum agents (azacitidine, vorinostat, panobinostat, chidamide) with limited efficacy and substantial toxicity, and next-generation selective inhibitors (valemetostat, BET proteolysis-targeting chimeras (ROTACs), valemetostat) with improved selectivity. **(B)** Rational combination of epigenetic priming with immunotherapy targets three convergent modules: restoring MHC-I antigen presentation, expanding the Tpex pool, and remodeling the immunosuppressive tumor microenvironment (TME). **(C)** Genetic and epigenetic engineering of immune memory cells. Genetic editing (left) employs CRISPR-Cas9 knockout or lentiviral overexpression of memory-associated transcription factors (FOXO1, c-Jun, NR4A, DNMT3A/TET2/ASXL1, BATF + IRF4) to indirectly remodel the epigenome. True epigenetic editing (right) utilizes locus-specific chromatin modifiers without altering DNA sequences, including dead Cas9 (dCas9)-KRAB for silencing, dCas9-p300 for activation, CRISPRon for targeted upregulation, and RENDER (Robust ENveloped Delivery of Epigenome-editor Ribonucleoproteins) for transient, non-integrating delivery. Transient mRNA or ribonucleoprotein (RNP) delivery offers enhanced safety; persistent lentiviral (LV) or adeno-associated viral (AAV) delivery ensures durable modification. OE, overexpression; KO, knockout.

**Table 2:** Summary table of epigenetic drugs/editing strategies in cancer.

Target	Tool/agent	Tumor type	Development stage	Efficacy & toxicity	Reference (article title + URL)
DNMT1/3A/3B	Azacitidine (5-Aza)	Acute myeloid leukemia (AML), MDS	FDA approved (2004)	Efficacy: restores silenced tumor suppressors. Toxicity: myelosuppression, gastrointestinal toxicity, injection site reaction.	Article: Epigenetic drugs in cancer therapy: mechanisms, immune modulation, and therapeutic applications URL: https://pmc.ncbi.nlm.nih.gov/articles/PMC12675902/
DNMT1/3A/3B	Decitabine	AML, myelodysplastic syndrome (MDS)	FDA approved (2006)	Efficacy: improves transfusion independence in MDS; synergizes with immunotherapy. Toxicity: severe neutropenia, thrombocytopenia, fatigue.	Article: Precision epigenetic therapies in oncology URL: https://pmc.ncbi.nlm.nih.gov/articles/PMC12446134/
Pan-HDAC (Class I/II)	Vorinostat (SAHA)	Cutaneous T-cell lymphoma (CTCL)	FDA approved (2006)	Efficacy: enhances anti-PD1 efficacy in solid tumors. Toxicity: diarrhea, fatigue, thrombocytopenia, QT prolongation risk.	Article: Epigenetic modifications: Key players in cancer heterogeneity and drug resistance URL: https://pmc.ncbi.nlm.nih.gov/articles/PMC10654239/
HDAC1/2	Romidepsin	CTCL, peripheral T-cell lymphoma (PTCL)	FDA approved (2009)	Efficacy: ORR >34% for refractory PTCL. Toxicity: nausea, lymphopenia, electrolyte disturbance.	Article: Precision epigenetic therapies in oncology URL: https://pmc.ncbi.nlm.nih.gov/articles/PMC12446134/
EZH2 (PRC2)	Tazemetostat	Epithelioid sarcoma, follicular lymphoma	FDA approved (2020)	Efficacy: durable tumor regression in EZH2-mutant B-cell lymphoma. Toxicity: fatigue, anemia, upper respiratory tract infection; secondary malignancy risk warning.	Article: Epigenetic drugs in cancer therapy URL: https://pmc.ncbi.nlm.nih.gov/articles/PMC11865116/
EZH1/EZH2	Tulmimetostat (CPI-0209)	ARID1A-mutant solid tumors, ovarian cancer	Phase I/II clinical trial	Efficacy: stronger H3K27me3 suppression vs tazemetostat; single-agent anti-tumor activity in ARID1A loss tumors. Toxicity: reversible cytopenias.	Article: Epigenetic Therapies in Endocrine-Related Cancers URL: https://pmc.ncbi.nlm.nih.gov/articles/PMC12345763/
DOT1L	Pinometostat (EPZ-5676)	KMT2A-rearranged acute leukemia	Phase I completed	Efficacy: transient clinical responses in pediatric/adult MLL-r leukemia; limited single-agent activity. Toxicity: fatigue, hepatotoxicity.	Article: Current Approaches to Epigenetic Therapy URL: https://pmc.ncbi.nlm.nih.gov/articles/PMC10594535/
LSD1 (KDM1A)	DC551040	AML, myelodysplastic syndrome	Phase I clinical trial	Efficacy: potent anti-proliferation in AML xenograft models; induces myeloid differentiation. Toxicity: mild liver enzyme elevation in preclinical models.	Article: A novel irreversible LSD1 inhibitor induces differentiation and exerts anti-leukemia activity in acute myeloid leukemia URL: https://www.nature.com/articles/s41392-026-02637-0
Menin-KMT2A interaction	Revumenib	Relapsed/refractory KMT2A-rearranged AML	FDA approved (2024)	Efficacy: ~53% composite complete remission rate in heavily pretreated MLL-r AML. Toxicity: differentiation syndrome, QT prolongation.	Article: Epigenetic drugs in cancer therapy: mechanisms, immune modulation, and therapeutic applications URL: https://pmc.ncbi.nlm.nih.gov/articles/PMC12675902/
Oncogene promoter (MYC)	dCas9-KRAB (DNA hypermethylation editor)	Non-small cell lung cancer (NSCLC), Hepatocellular carcinoma (HCC)	Preclinical (in vitro + mouse xenograft)	Efficacy: site-specific MYC silencing; robust suppression of tumor proliferation in vivo. Toxicity: LNP/AAV delivery-related mild inflammation; minimal off-target methylation vs systemic small molecules.	Article: Epigenome editing based treatment: Progresses and challenges URL: https://pmc.ncbi.nlm.nih.gov/articles/PMC12925790/
Tumor suppressor promoter (miR-200c)	dCas9-TET1 (DNA demethylation editor)	Triple-negative breast cancer (TNBC)	Preclinical (cell lines)	Efficacy: reactivate silenced miR-200c, inhibit EMT and metastasis. Toxicity: low global epigenome disturbance; risk of aberrant activation of oncogenic loci at high dosage.	Article: CRISPR/dCas9-TET1–mediated epigenetic editing reactivates miR-200c in breast cancer cells URL: https://www.pmop.cn/pubmed/40877540
KDM4A gene locus	CRISPRoff (dCas9-DNMT3A-KRAB) + KDM4 inhibitor QC6352	Colon cancer, breast cancer, HCC	Preclinical (cell lines)	Efficacy: dual blockade of KDM4 transcription + protein activity; prevents adaptive drug resistance seen with single small-molecule treatment. Toxicity: combinatorial treatment shows no additive cytotoxicity to normal epithelial cells.	Article: Epigenetic editing and epi-drugs: a combination strategy to simultaneously target KDM4 as a novel anticancer approach URL: https://pmc.ncbi.nlm.nih.gov/articles/PMC12177974/

### 5.1 Small-molecule epigenetic inhibitors

Epigenetic-targeted strategies can be categorized into two broad classes: (1) broad-spectrum chromatin modifiers that globally alter chromatin accessibility, and (2) lineage-specific transcriptional regulators that selectively modulate exhaustion-associated gene networks.

Drugs targeting DNMTs, HDACs, and histone methyltransferases have shown preclinical and early clinical potential in reversing exhaustion programs and enhancing antitumor immunity. However, first-generation epigenetic agents have encountered clinical setbacks. As noted in Section 4.2, in older patients with AML, azacitidine monotherapy is not more effective than conventional care regimens at maintaining event-free survival (121). In solid tumors, broad-spectrum HDAC inhibitors such as vorinostat and panobinostat have demonstrated the limited effectiveness of single agents and their substantial dose-limiting toxicities (including myelosuppression, fatigue, and gastrointestinal events), restricting their integration with immunotherapy (122). These failures highlight a central translational bottleneck: the narrow therapeutic window of non-selective epigenetic drugs necessitates biomarker-driven patient selection and mechanism-based combination strategies.

Dual DNMT/HDAC inhibitors remodel the immune microenvironment of colorectal cancer, enhance anti-PD-L1 effectiveness, increase CD8+ T-cell infiltration, reduce Tregs, and favor the formation of memory T cells (134). Bifunctional HDAC/DNMT inhibitors induce viral mimicry (derepression of endogenous retroviral elements triggering type I interferon responses), activate STING-dependent signaling, and sensitize tumors to immunotherapy in triple-negative breast cancer (135, 136). Dual poly ADP ribose polymerase (PARP)/HDAC inhibitors elicit intratumoral STING-mediated antitumor immunity (137).

Lineage-specific transcriptional regulators provide a more targeted approach. Preclinical models demonstrate that targeting ETV7, Id2, BLIMP1, and NR4A3 can restore memory potential (138–140). However, clinical translation is limited by systemic toxicity and lack of cell-type specificity. PROTACs induce targeted protein degradation through E3 ligase recruitment and proteasomal degradation; preclinical studies show that BET-targeting PROTACs such as ARV-771 effectively deplete BRD4, suppress oncogenic transcription, and inhibit tumor growth in castration-resistant prostate cancer and haematological malignancies (141), although *in vivo* delivery and off-tumor toxicity in immune cells remain a challenge. Achieving cell-type specificity while minimizing toxicity is a central challenge.

Recently developed highly selective epigenetic inhibitors have improved therapeutic windows. Compared with conventional small-molecule inhibitors, PROTAC degraders achieve more complete target blockade and a wider therapeutic window via an event-driven, catalytic mechanism of action. Furthermore, exploiting differential E3 ubiquitin ligase expression between normal and diseased tissues—exemplified by the low expression of VHL in platelets that spares BCL-xL degradation by the PROTAC DT2216—can reduce on-target toxicity in normal cells and improve the therapeutic index (141). Weiss et al. showed that deletion of the exhaustion-associated PD-1 enhancer lowers PD-1 expression to a functional “sweet spot” in exhausted CD8^+^ T cells, which augments their antiviral efficacy while sparing the host from the severe immunopathology seen upon total PD-1 loss (142). This nuanced approach preserves sufficient PD-1 signaling to reduce the risk of autoimmunity while restoring effector capacity. Additionally, phase 2 trials have shown that the dual EZH1/EZH2 inhibitor, valemetostat (DS-3201b), has potential for treating relapsed or refractory adult T-cell leukemia/lymphoma, with an overall response rate of 48.0% and manageable safety profile (143). Preclinical studies have shown that tazemetostat upregulates MHC class I and adhesion molecules to enhance CAR T-cell therapy, and valemetostat (dual EZH1/EZH2 inhibitor) also demonstrates synergistic anti-tumor efficacy with CAR-T cells in multiple models (144).

Although first-generation epigenetic inhibitors face limitations in specificity and toxicity, emerging strategies, including PROTAC-mediated degradation, allosteric modulation, and locus-specific epigenetic tuning, promise to overcome these barriers and achieve cell-type-selective chromatin remodeling.

### 5.2 Rational combination of epigenetic modifiers with immunotherapy

Epigenetic modifiers enhance immunotherapy through three convergent mechanisms: restoring MHC class I expression and antigen presentation, expanding the stem-like memory T-cell pool, and remodeling the immunosuppressive TME.

As described in Section 5.1, dual DNMT/HDAC inhibitors enhance the effectiveness of PD-L1 inhibitors in colorectal cancer (134). EZH2 inhibition activates the dsRNA-STING-interferon axis and sensitizes prostate cancer to PD-1 blockade (145). EZH2 inhibition also enhances PD-L1 stability in colorectal cancer via USP22-mediated deubiquitination (146). BET inhibition sensitizes resistant melanoma to anti-CTLA-4 (147), enhances the effectiveness of BCG vaccine (148), and combines with SMAC mimetics to restrict tumor growth (149). TLR7/8 agonists combined with epigenetic inhibitors suppress triple-negative breast cancer (150).

Clinical trials are beginning to validate these combinations. The phase II CAPability-01 trial showed that in colorectal cancer chidamide, sintilimab, and bevacizumab achieved an objective response rate (ORR) of 44.0% and median progression-free survival (PFS) of 7.3 months in the triple-therapy arm, compared with 13.0% ORR and 1.5 months median PFS in the dual-therapy (sintilimab plus chidamide) arm (151). This triple-combination therapy is a substantial improvement over standard-of-care chemotherapy which typically achieves a median PFS of 2–3 months in refractory in microsatellite stable (MSS)/mismatch repair proficient (pMMR) metastatic colorectal cancer. Bevacizumab contributes to this effectiveness through vascular normalization, which improves T cell infiltration and reduces immunosuppressive myeloid cell accumulation in the TME (151). Low-dose decitabine priming prior to anti–PD-1 therapy promotes the expansion and sustained activation of CD8+ progenitor exhausted T cells, thereby augmenting antitumor immunity (120). Trials comparing sequential versus concurrent administration are needed to define optimal protocols.

Emerging evidence suggests that the relative timing of epigenetic priming and immunotherapy administration critically influences the therapeutic outcomes. Decitabine pretreatment followed by PD-1 blockade expands the Tpex pool and enhances antitumor responses in preclinical models (120). Conversely, concurrent administration of HDAC inhibitors with checkpoint blockade might increase toxicity without proportional gains in effectiveness. Therefore, biomarker-driven temporal optimization is needed in clinical trial design.

Combination therapies are more effective than single-agent epigenetic inhibitors because they simultaneously target tumor-intrinsic pathways and immune cell states. This rational integration is the most promising near-term approach for clinical translation, although biomarker-guided patient selection and temporal optimization are critical unresolved questions.

### 5.3 Genetic and epigenetic engineering of immune memory cells

Strictly defined, epigenetic editing refers to locus-specific modification of chromatin states (using tools such as dCas9-KRAB or dCas9-p300) without altering DNA sequences. By contrast, most current cancer immunotherapy studies use genetic editing (CRISPR-Cas9 knockout or lentiviral overexpression) to indirectly remodel the epigenome. These two approaches differ fundamentally in mechanism, reversibility, and regulatory considerations, and require clear differentiation both in terms of terminology and experimental design.

Current studies predominantly use genetic manipulation to indirectly achieve epigenetic remodeling. Genetic editing of memory-associated genes has emerged as a powerful strategy. Knockout of DNMT3A, TET2, or ASXL1 preserves the Tpex population under chronic antigen exposure; FOXO1 overexpression induces memory transcriptional programs and enhances CAR T-cell persistence (152, 153); c-Jun overexpression induces exhaustion resistance (154); NR4A knockout enhances memory potential (111); BATF and IRF4 cooperate to counteract exhaustion (119); and BLIMP1 and NR4A3 bidirectionally regulate stemness (140).

True epigenetic editing (locus-specific chromatin modification without DNA sequence alteration) remains largely at the preclinical stage; however, recent breakthroughs have accelerated its translational potential. Targeted demethylation of the TCF7 promoter or enhancer activation could theoretically restore memory potential, but such tools have not been adequately validated in cancer models. In 2025, Goudy et al. (155) reported the development of an all-RNA platform for multiplexed epigenetic programming in primary human T cells using CRISPRoff and CRISPRon editors. This is a major advance. CRISPRoff-mediated gene silencing is maintained through numerous cell divisions, T-cell stimulations, and *in vivo* adoptive transfer, avoiding cytotoxicity or chromosomal abnormalities inherent to multiplexed Cas9-mediated genome editing (155). The authors successfully combined genetic and epigenetic engineering using orthogonal Cas12a-dCas9 systems for targeted CAR knock-in at the TRAC locus and CRISPRoff silencing of RASA2, a molecular brake on T-cell activation. In NSG mice bearing Nalm6 leukemia, RASA2-silenced CAR T-cells showed better tumor control and extended survival than that of control CAR T-cells. These findings establish a GMP-compatible framework for clinical translation; however, no epigenetic editing strategies have yet entered clinical trials for CAR T-cell therapy.

Recent advances in epigenome editing have yielded promising tools for clinical translation. The CRISPRoff system enables heritable gene silencing through DNA methylation without inducing double-strand breaks, offering a safer alternative to conventional gene knockout (156). Its reversibility depends on targeted demethylation mechanisms (such as CRISPRon or TET-based approaches) and is not spontaneous; therefore, “reversible” refers to engineered reversibility rather than natural turnover. Additionally, transient expression of epigenetic modifiers via mRNA electroporation allows temporary chromatin remodeling without permanent genetic alteration, potentially reducing long-term safety risks. Robust ENveloped Delivery of Epigenome-editor Ribonucleoproteins (RENDER), a platform for transient, non-integrating delivery of programmable epigenetic modulators into human cells, has achieved durable transcriptional modulation of endogenous targets such as CD55 and CD81 in donor-derived primary T cells, with gene repression persisting beyond the detectable presence of editing complexes (157). These technologies bridge the gap between genetic and epigenetic engineering by providing locus-specific, reversible chromatin control with reduced genotoxicity.

Safety concerns for epigenetic editing include off-target chromatin modifications, long-term stability of engineered states, and immunogenicity of Cas proteins. A critical distinction of epigenetic editing from genetic editing is the absence of insertional mutagenesis risk with transient mRNA delivery; however, lentiviral delivery of dCas9 fusions retains the risk of semi-random integration. Both transient mRNA electroporation and RNP delivery (like RENDER) enable durable epigenetic silencing without sustained editor expression, thereby minimizing off-target risks and immunogenicity associated with prolonged Cas activity (155, 157). In contrast, persistent expression platforms such as AAV or lentivirus ensure continuous editor presence but may increase the risk of prolonged off-target epigenetic modifications and raise additional safety concerns, including viral integration and host immune responses. Regulatory frameworks for epigenetic editing still need to be developed as current guidelines primarily address nuclease-based genome editing.

In summary, although genetic editing of memory-associated transcription factors has been shown to be effective in preclinical studies, true epigenetic editing, enabling locus-specific chromatin modification without DNA sequence alteration, is still in the early phases of development and remains the frontier for precision immune engineering. Distinguishing between genetic and epigenetic editing approaches is essential for accurate scientific communication and regulatory clarity.

## 6 Current challenges and future perspectives

Although the epigenetic mechanisms underlying tumor immunological memory and related targeted strategies have achieved phased advances, substantial bottlenecks still remain (158, 159). Significant gaps between basic research and clinical translation persist in mechanistic understanding, technical systems and clinical practice. Addressing these limitations is critical to guiding future research and translational applications (160, 161).

### 6.1 Key challenges in epigenetic reprogramming of immunological memory in cancer

Research on the epigenetic mechanisms of tumor immunological memory has made gradual progress; nevertheless, prominent gaps still exist in mechanistic interpretation, technical systems and clinical translation, which urgently need to be resolved (162).

Mechanistic level: The epigenetic regulation of tumor immunological memory still faces many unresolved challenges, and its core regulatory networks remain poorly defined (163). DNA methylation, histone modification, non-coding RNAs and 3D genome remodeling are investigated in a fragmented manner with ambiguous functional boundaries and extensive regulatory redundancy (61, 66, 164). Single-node interventions are easily compensated by alternative regulatory circuits, thereby impairing immune memory homeostasis (164). Exhaustion-associated epigenetic imprints are inheritable and highly stable (165). As a key transcription factor that maintains chromatin accessibility in exhausted T cells, TOX forms a persistent epigenetic barrier, locks T cells into an exhausted cell fate and renders them resistant to conventional immunotherapeutic clearance (89, 118). The abundance of TCF1+ stem-like T cells serves as a reliable predictor for immune checkpoint blockade efficacy, while this cell population is negatively programmed by tumor microenvironments (166, 167). Spatiotemporal heterogeneity is still insufficiently characterized: CD8+ T cells undergo hierarchical differentiation from stem-like progenitors to terminal exhaustion, accompanied by altered TAD boundaries and progressive loss of chromatin loops, which endow T cell subpopulations with divergent epigenetic landscapes (168, 169). Metabolic-epigenetic coupling has become an emerging research hotspot. Histone lactylation bridges metabolic signals and epigenetic regulation to drive immunosuppressive programs; however, the upstream and downstream signaling cascades as well as core regulatory axes still require further clarification (65).

Clinical intervention level: Major epigenetic inhibitors targeting DNMT, HDAC and BET lack cell- and tissue-specific targeting specificity, frequently leading to off-target effects as well as hematological and gastrointestinal toxicities (170). In phase I/II trials of the BET inhibitor molibresib for solid tumors, 98% of patients suffered from adverse events, and 19% permanently discontinued treatment due to severe toxicity, which greatly limits its clinical applicability (171). More importantly, these agents cannot precisely distinguish immunostimulatory cell subsets from immunosuppressive ones (148). Accumulated evidence demonstrates that epigenetic drugs remodel the tumor epigenome while simultaneously regulating immunosuppressive compartments such as myeloid-derived suppressor cells. Different inhibitors exert divergent effects on regulatory T cells and myeloid suppressor populations, resulting in complex bidirectional immune outcomes and unpredictable clinical efficacy (172). Tumor cells can actively establish inflammation-related epigenetic memory, which persistently drives T cell dysfunction and induces primary and acquired resistance to immunotherapy (173, 174). For instance, chronic antigen stimulation sustains high TOX expression, locks the exhaustion-specific chromatin accessibility landscape, and maintains the exhausted phenotype even after T cells escape from the tumor microenvironment (27). Current intervention strategies are incapable of erasing such intrinsic immune evasion memory, highlighting an urgent demand for next-generation targeted tools with both cell-selectivity and epigenetic remodeling capacity (175, 176).

Technical application level: Traditional bulk sequencing cannot resolve the subpopulation heterogeneity of immune memory cells, masking subtype-specific epigenetic signatures. Although single-cell epigenetic profiling enables the identification of such heterogeneity, its wide application in large-scale clinical cohorts is restricted by high costs, low throughput and the lack of standardized workflows (177). Breakthrough progresses have been made in 3D genome reprogramming research. During the differentiation of exhausted CD8^+^ T cells, the transcription factor IRF8 recruits CTCF to facilitate chromatin loop formation, modulates enhancer–promoter spatial proximity, and further controls the transcription of exhaustion-related genes (164). These findings confirm that 3D genome reorganization acts as a core regulatory mechanism of T cell exhaustion, while its potential as a clinical intervention target remains to be validated (178). Meanwhile, clinical practice lacks a standardized detection system for epigenetic biomarkers. Preoperative radiotherapy and chemotherapy can induce aberrant genome-wide methylation and chromatin remodeling. For example, platinum-based agents sustain elevated methylation of the STING1 promoter for months, confounding the interpretation of immune memory-related epigenetic signatures (179). Although cfDNA methylation profiling and single-cell epigenomics show great potential for dynamic therapeutic monitoring, their standardization and clinical validation are still in the early stage, substantially reducing the accuracy of efficacy prediction and patient stratification (180, 181).

### 6.2 Future prospects

Given these persistent challenges, future epigenetic research on tumor immunological memory must undergo three core paradigm shifts: from reversing terminal exhaustion to locking stem-like memory phenotypes, from targeting a single epigenetic pathway to intervening hub nodes in metabolic-epigenetic networks, and from static biomarker detection to dynamic monitoring-adaptive intervention closed-loop (182). In response, we believe that breakthrough explorations along these three dimensions will drive future advances. Locking stem-like memory phenotypes is one of the most translationally promising strategies at present. Terminally exhausted T cells undergo irreversible TAD remodeling and loss of enhancer-promoter loops; forced reversal of the exhausted state yields limited efficacy and may increase the risk of genomic instability. Conversely, TCF1+ stem-like memory T cells maintain a plastic chromatin state under chronic antigen stimulation, and their abundance reliably predicts immunotherapy response (117). We propose that next-generation epigenetic intervention strategies should focus more on epigenetic locking via CRISPR-Cas9 system to stably activate stemness-related genes while repressing exhaustion-driven factors in stem-like T cells, thereby locking cell fate at the memory progenitor stage. Knockout of DNMT3A/TET2/ASXL1 extends T cell *in vivo* persistence to more than one year; dCas9-KRAB targeting the PDCD1 promoter achieves durable gene silencing of over 90% (62). Future development of multi-gene epigenetic programming systems could preinstall anti-exhaustion epigenetic signatures in CAR-T/TCR-T cells to sustain long-term immune memory function in solid tumor microenvironments.

Hub nodes in metabolic-epigenetic networks are more valuable for intervention than single signaling pathways, and a dynamic monitoring-adaptive intervention closed-loop is viable for precision immunotherapy (65). Lactate upregulates PD-1 via H3K18la, remodels the glioma immune microenvironment in a CBX3-dependent way, and upregulates ACAT2 to form a positive feedback loop regulating cholesterol metabolism and histone acetylation (183). We postulate that future interventions targeting hub molecules at these network crosstalk nodes, including PROTAC-mediated ACAT2 degradation and LDHA inhibitor treatment, have the potential to abrogate lactate production, histone lactylation and downstream lipid metabolic reprogramming (184). Meanwhile, artificial intelligence-assisted metabolic modeling is expected to predict the systemic effects of such metabolic interventions, thereby helping to circumvent treatment failure caused by pathway compensation (185). Currently, most clinical epigenetic biomarkers are static, failing to capture dynamic epigenetic changes in immune memory cells during treatment (174). To tackle this limitation, future studies may combine cell-free DNA methylation profiling, single-cell epigenomics and AI algorithms to develop patient-specific epigenetic clocks, allowing real-time tracking of the TCF1^+^ memory T cell/exhausted T cell ratio and dynamic methylation of critical promoters (186). Future integration of an inducible safety switch into the closed-loop system may facilitate a monitoring-alert-intervention-pause framework, while extended follow-up (≥15 years) could enable systematic evaluation of secondary malignancy risks. However, clinical translation still faces technical and safety challenges, and the long-term benefit–risk balance awaits validation in prospective cohort studies. In summary, exploring the three strategies-locking stem-like phenotypes, targeting network hub nodes, and constructing closed-loop dynamic regulation-may gradually promote precise epigenetic modulation of tumor immunological memory, advancing immunotherapy from empirical application to a controllable, precise paradigm.

## 7 Discussion

Epigenetic rewiring is emerging as an important organizing principle that links immunological memory, tumor immune escape, and therapeutic response. Rather than being merely a passive imprint of chronic antigen exposure, epigenetic rewiring actively biases antitumor immunity toward durable memory, exhaustion, or pathological tolerance. Across adaptive and innate immune compartments, DNA methylation, histone modification, ncRNAs, chromatin remodeling, and three-dimensional genome architecture jointly define the transcriptional potential, persistence, and recall capacity of memory immune cells (187–189). In cancer, the TME imposes persistent metabolic, inflammatory-like, and immunosuppressive pressures that reshape these epigenetic programs, thereby impairing memory T- and B-cell differentiation, inducing dysfunctional trained immunity in myeloid and NK cells, and disrupting the coordinated crosstalk between innate and adaptive immune memory networks (190, 191). Importantly, immune memory should no longer be viewed as a fixed cellular state, but as a dynamic and therapeutically adjustable epigenetic continuum. This perspective may help explain why some patients derive durable long-term benefit from immunotherapy, whereas others develop primary or acquired resistance despite the presence of some degree of immune infiltration or an initial therapeutic response. However, clinical translation of epigenetic rewiring remains limited by the lack of standardized definitions, suitable biomarkers, cell-type-specific epigenetic interventions, and clinically scalable approaches capable of resolving the spatial and cellular heterogeneity of immune memory (13, 192). Thus, durable antitumor immunity may require not only reinvigoration of exhausted effector cells, but also restoration of a stable, spatially organized, and metabolically compatible memory-competent epigenetic landscape.

Future studies should move beyond single-marker or single-cell-subset analyses and establish integrated “epigenetic memory atlases” that combine chromatin accessibility, DNA methylation, histone modification, transcriptomic, metabolic, and spatial information according to the disease stage and treatment course. Therapeutically, the next generation of strategies should shift from broad epigenetic inhibition toward programmable and context-adaptive epigenetic modulation, including optimized low-dose priming, cell-selective delivery systems, CRISPR-based epigenetic editing, metabolic-epigenetic co-targeting, and AI-guided patient stratification. A particularly important future direction is to distinguish reversible dysfunctional plasticity from irreversible immune exhaustion, as this boundary may determine whether immune memory can be functionally restored through pharmacological priming or epigenetic editing, or whether it must be rebuilt using adoptive cell therapy and engineered immune cells. Ultimately, transforming epigenetic rewiring from a mechanism of tumor immune escape into a controllable therapeutic vulnerability may provide a foundation for more durable, precise, and memory-directed cancer immunotherapy.
